# Zoonoses in the Bedroom

**DOI:** 10.3201/eid1702.101070

**Published:** 2011-02

**Authors:** Bruno B. Chomel, Ben Sun

**Affiliations:** Author affiliations: University of California, Davis, California, USA (B.B. Chomel);; California Department of Public Health, Sacramento, California, USA (B. Sun)

**Keywords:** Zoonoses, dog, cat, kissing, sleeping, licking, bacteria, viruses, parasites, perspective

## MEDSCAPE CME

Medscape, LLC is pleased to provide online continuing medical education (CME) for this journal article, allowing clinicians the opportunity to earn CME credit.

This activity has been planned and implemented in accordance with the Essential Areas and policies of the Accreditation Council for Continuing Medical Education through the joint sponsorship of Medscape, LLC and Emerging Infectious Diseases. Medscape, LLC is accredited by the ACCME to provide continuing medical education for physicians.

Medscape, LLC designates this Journal-based CME for a maximum of 1 *AMA PRA Category 1 Credit(s)*^TM^. Physicians should claim only the credit commensurate with their participation in the activity.

All other clinicians completing this activity will be issued a certificate of participation. To participate in this journal CME activity: (1) review the learning objectives and author disclosures; (2) study the education content; (3) take the post-test and/or complete the evaluation at www.medscapecme.com/journal/eid; (4) view/print certificate.

**Release date: January 26, 2011; Expiration date: January 26, 2012**

## Learning Objectives

Upon completion of this activity, participants will be able to:

- Describe transmission of plague by pets living in very close contact to their owners
- Describe P*asteurella* spp. infections transmitted from pets to their owners
- Describe parasitic zoonoses associated with pet ownership

## Editor

**P. Lynne Stockton, VMD, MS, ELS(D), **Technical Writer-Editor, *Emerging Infectious Diseases. Disclosure: P. Lynne Stockton, VMD, MS, ELS(D), has disclosed no relevant financial relationships.*

## CME AUTHOR

**Laurie Barclay, MD**, freelance writer and reviewer, Medscape, LLC. *Disclosure: Laurie Barclay, MD, has disclosed no relevant financial relationships.*

## AUTHORS

*Disclosures:* ***Bruno B. Chomel, DVM, PhD;*** *and* ***Ben Sun, DVM, MPVM*** *, have disclosed no relevant financial relationships.*
