# Supplementary material for: Zoonoses in the Bedroom
Source: Emerg Infect Dis. 2011 Feb;17(2):167–72. doi: 10.3201/eid1702.101070 (PMC3298380; doi:10.3201/eid1702.101070)
Supplement: Related texts

# Review article: Zoonoses in the Bedroom

**DOI:** 10.3201/eid1702101070

**Authors:** Bruno B. Chomel, Ben Sun

**Affiliations:** Author affiliations: University of California, Davis, California, USA (B.B. Chomel);; California Department of Public Health, Sacramento, California, USA (B. Sun)

## Abstract

In most industrialized countries, pets are becoming an integral part of households, sharing human lifestyles, bedrooms, and beds. The estimated percentage of pet owners who allow dogs and cats on their beds is 14%–62%. However, public health risks, including increased emergence of zoonoses, may be associated with such practices.

As modern society is becoming more urbanized, the presence in our households of traditional pets, or even exotic creatures, is increasing in popularity. Pets have become an integral part of the family and are often considered to be extended family (*1*). Having pets brings many benefits, such as psychological support, friendship, and even good health practices (exercising or reducing stress) (*1*). However, in many countries, pets have become substitutes for childbearing and child care, sometimes leading to excessive pet care. For example, one of the most recent trends in pet care in Asia is hair dyeing. A recent news story claims, “Dyeing pets is popular in many developed countries like Japan and Korea, but China is quickly catching on” (www.wibw.com/home/headlines/101783553.html [cited 2010 Nov 29]). Not only are pets present in our daily environment, but they have also conquered our bedrooms. Sharing our resting hours with our pets may be a source of psychological comfort, but because pets can bring a wide range of zoonotic pathogens into our environment, sharing is also associated with risks.

## Sleeping with “Man’s Best Friends”

In the United States, >60% of households have pets (*2*); pet ownership increased from 56% in 1988 to 62% in 2008 (www.americanpetproducts.org/press_industrytrends.asp [cited 2010 Jun 23]). Among dog owners, 53% consider their dog to be a member of the family. A surprising 56% of dog owners sleep with their dog next to them; ≈50% of dogs sleep on the bed. Among dogs that sleep with their owners, 62% are small dogs, 41% are medium sized, and 32% are large (http://pets.webmd.com/features/pets-in-your-bed [cited 2010 Jun 23]). In a 2005 survey about dog ownership conducted by the American Kennel Club, 21% of dog owners interviewed said that they slept with their dog regularly; women were more likely than men to allow the practice (25% to 16%) (www.akc.org/pdfs/press_center/press_releases/2006/ValentineSurvey.pdf [cited 2010 Nov 29]). Another 16% said that their dogs snuck into their beds at least once in a while (Sacramento Bee, April 9, 2006, L1–L2). Among cats, 62% slept with their adult owners and another 13% slept with children.

In the United Kingdom, an estimated 6.5 million dogs live in ≈25% of households (*3*). In a survey of 260 dog-owning households in a community in Cheshire, 19% of the dogs were sleeping on the bedroom floor and 14% on their owner’s bed (*3*). A survey conducted in 1995 with regard to cats >12 years of age throughout the United Kingdom found that among 1,236 of these older cats, 45% were sleeping regularly on the owner’s bed (www.fabcats.org/behaviour/understanding/oldcats.html [cited 2010 Jun 23]).

In the Netherlands, the pet population is ≈2 million dogs and 3 million cats (*1*). The percentage of households with pets increased from 50% in 1999 to 55% in 2005. A recent study indicated that among 159 households with pets, 50% of pet owners interviewed allowed the pet to lick their face; 60% of pets visited the bedroom; 45% of dogs and 62% of cats were allowed on the bed; and 18% and 30% of the dogs and cats, respectively, were allowed to sleep with the owner in bed (*1*).

In France, the estimated pet population is ≈8.1 million dogs in 25% of households and ≈9 million cats in 26% of households. The number of dogs increased from ≈4 million in the late 1950s to its current 8.1 million (www.naturanimal.com/chiens/pratique/chiffres-chiens.php [cited 2010 Nov 29]). Le Monde in 2002 reported that ≈45% of cat owners and ≈30% of dog owners slept with their pet (www.lemonde.fr/cgi-bin/ACHATS/acheter.cgi?offre=ARCHIVES&type_item=ART_ARCH_30J&objet_id=785025 [cited 2010 Jun 23]).

Although such trends (Table 1) should be considered with some caution because they were obtained from media sources and may not accurately reflect the true prevalence of this behavior, the zoonotic disease risks associated with such behavior should be evaluated on the basis of the scientific literature. We therefore searched PubMed for any peer-reviewed publication that clearly documented human exposure to zoonotic diseases by sleeping with, sharing a bed with, kissing, or being licked by pets.

**Table 1:** Estimated pet dog and cat populations in developed countries and estimated percentage of these pets sleeping on/in owner’s bed , 1974–2010

Country	Dogs			Cats	
	Estimated population, millions	% Sleeping on/ in owner’s bed		Estimated population, millions	% Sleeping on/ in owner’s bed
United States	60	21–33		75	60
United Kingdom	8	14		8	45
France	8	30		9	45
The Netherlands	2	45		3	62

## Bacterial, Parasitic, and Viral Zoonoses

### Plague

During a 1974 outbreak of plague in New Mexico, USA, 7 cases of bubonic plague were investigated. One patient noticed flea bites the morning after he allowed his flea-infested cat to share his bed (*4*). Similarly, in a series of 23 cases of plague related to cat exposure, a 9-year-old boy from Arizona had handled and slept with a sick cat (*5*). Another case, which occurred in 1983 in New Mexico, was likely acquired after indoor/outdoor cats slept with the patient (*6*). More recently, a 2008 matched case–control study (*7*) surveyed 9 plague survivors, 12 household members of these survivors, and 30 age- and neighborhood-matched controls about household and individual exposures. Four (44%) survivors and 3 (10%) controls (matched odds ratio 5.7, 95% confidence interval [CI] 1.0–31.6) reported sleeping in the same bed with a pet dog, which remained significantly associated with infection in a multivariate logistic regression model (p = 0.046). Such behavior is of concern because dogs may facilitate transfer of infected fleas into the home and, unlike cats, rarely show clinical signs of infection that could serve as a warning.

### Chagas Disease

A study in northwest Argentina showed that dogs and cats infected with the Chagas disease agent, *Trypanosoma cruzi*, increased risk for domestic transmission of *T. cruzi* to the Chagas disease vector, *Triatoma infestans* bugs (*8*). Infection rates were significantly higher when infected dogs shared sleeping areas with humans than when they did not (relative risk 1.79; 95% CI 1.1–2.91).

### Cat-Scratch Disease

Cat-scratch disease is mainly transmitted to humans when they are scratched by a cat that harbors *Bartonella henselae*–infected fleas and flea feces (*9*). However, a few documented cases have been associated with sleeping or being licked by a household pet. For example, a systemic case of cat-scratch disease with hepatic, splenic, and renal involvement caused by *B. henselae* was diagnosed by immunofluorescence assay, PCR, computed tomography, and histologic examination. The patient was a 9-year-old aboriginal girl from Taiwan, who had been sleeping with a cat at night (*10*). In addition, *B.* *hensalae* infection was suspected and confirmed by serologic testing of a 50-year-old man from Japan, who had left cervical lymphadenopathy and owned a dog that often licked his face (*11*). In a study of risk factors associated with cat-scratch disease in Connecticut, USA, case-patients were more likely than matched controls to have been scratched or bitten by a kitten, licked on the face by a kitten, slept with a kitten, or combed a kitten (*12*).

### *Pasteurella* spp. and *Capnocytophaga* *canimorsus* Infections

Several reports describe human infections by *Pasteurella* spp. that were acquired after close contact with pets, including sharing a bed, being licked by, or kissing the pets. In 1985, a case of meningitis caused by *P. multocida* in a 60-year-old housewife living in the United Kingdom was reported (*13*). She admitted to regularly kissing the family dog. *P. multocida* isolates from buccal and nasal swabs of the dog were identical to isolates from the woman. Two cases of meningitis in newborn children (<1 month of age) have been reported; 1 was associated with a pet cat stealing a baby’s pacifier and using it as a toy, and the other was associated with a pet dog that often licked the baby’s face (*14*). Of 38 reported cases of *P. multocida* meningitis in infants, 27 (87%) of 31 infants that had been exposed to animals had been exposed directly or indirectly to the animals’ oropharyngeal secretions through licking or sniffing (*14*). A case of *P. multocida* infection of a hip replacement site occurred in a 69-year-old man (*15*). This man indicated that the dog had shared his bed before and after his operations, sleeping under the covers on the side of the affected leg, as it had done every night for the past 10 years (*15*).

Being licked by pets is a common source of human infection with *P. multocida* (*16**–**19*), but in a case described by Wade et al. (*16*), transmission to an infant occurred from another person. After the 2 family dogs had licked the hands of the infant’s 2-year-old brother, the older boy allowed the infant to suck on his little finger. Heym et al. (*18*) describe a case in France in which a total knee arthroplasty site became infected with *P. multocida* after the patient’s dog licked a small wound on the third toe of the leg that had been operated on. In another case, *P. multocida* was cultured from a wound abscess that developed in a 48-year-old obese woman 6 weeks after hysterectomy and panniculectomy for endometrial cancer (*20*); her cat had licked the wound. In France, meningitis caused by *P. multocida* developed in a 67-year-old patient with chronic, purulent otorrhea of the right ear. His dog frequently licked the patient’s right ear (*21*), and cultures from the dog's saliva also grew *P. multocida*. The isolates had identical biochemical patterns, and pulsed-field gel electrophoresis (PFGE) confirmed genotypic similarities. After digestion of genomic DNA with the infrequently cleaving restriction endonuclease *Sma*I, banding-pattern analysis showed clonal similarity between the isolates from the patient and the dog. In Japan, paranasal sinusitis caused by *P. multocida* was diagnosed for a 39-year-old woman with rhinorrhea and headache (*22*). The patient’s cat awakened her every morning by licking her. *P. multocida* isolates from the woman’s nasal discharge and the cat’s saliva were similar with respect to biochemical properties, serotype, and drug susceptibility.

Kissing pets can also transmit zoonoses. A study in Japan of 24 pet owners (11 cats and 3 dogs) found no *Pasteurella* spp. in the oral cavity of the 19 owners who had not kissed their cat, but isolated *P. stomatis* from the oral cavity of 1 of 2 owners who had kissed their cat and in 2 of 3 dog owners who had kissed their dog (*23*). Also in Japan, meningitis caused by *P. multocida* developed in a 44-year-old woman who admitted that she was regularly kissing the dog’s face and feeding it by transferring food mouth to mouth (*24*). As suggested by Kawashima et al. (*24*), “recent increase in pet ownership is likely to increase human exposure to *P. multocida.*” These authors identified at least 2 other cases of *P. multocida* meningitis between 2000 and 2010; these cases developed after the patients kissed a pet dog and a pet rabbit.

*Capnocytophaga canimorsus* infections in humans have been associated with being licked by or sleeping with a dog or cat. In Finland from 1988 through 1994, several cases of *C. canimorsus* septicemia were identified; 2 cases were associated with sleeping with and/or being licked by a pet (*25*). For an 81-year-old woman with cellulitis of the right leg and an ulcer between the fourth and fifth toe, *C. canimorsus* was isolated from a blood culture. This patient indicated that she slept with her cat in her bed and that the cat licked her feet and toes. A 60-year-old patient with chronic eczema died of septic shock and renal failure and disseminated intravascular coagulation caused by *C. canimorsus* (*25*). The ulcerous chronic eczema of his legs was the most probable port of entry for the organism because his dog used to lick his legs. In Kansas, USA, a splenectomized 44-year-old man died after infection with *C. canimorsus* (*26*). The man had lived in a trailer and collected scrap metal to sell; he had several cuts and scratches on his forearms and hands. His recently acquired German shepherd puppy reportedly licked the open abrasions on the man’s hands, but no bite was reported. In Australia, septicemia and multiorgan failure developed in a 48-year-old woman after her fox terrier puppy licked a minor burn wound on the top of her left foot (*27*).

### *Staphylococcus intermedius* Infections

*Staphylococcus intermedius* is a common commensal bacterium in dogs and cats and has rarely been identified as causing human infection (*28*). However, in Japan, *S. intermedius* developed in the mastoid cavity of a 51-year-old woman after mastoidectomy for chronic otitis media with cholesteatoma (*28*). Her dog had licked her ears, and bacterial strains from the dog’s saliva and the patient’s otorrhea were confirmed by PFGE to be identical. Similarly, a 28-year-old woman with a history of endoscopic pituitary adenoma resection reported 3 weeks of foul-smelling nasal discharge (*29*). Nasal endoscopy indentified a purulent sinus infection caused by methicillin-resistant *S. intermedius*. Cultures from the patient's pet bulldog also grew *S. intermedius* strains that were confirmed by PFGE to be identical to those of the patient. The patient reported having had close physical contact with her dog, including frequent licking of her face, and that the dog had recent bouts of pyoderma requiring treatment with antimicrobial drugs.

### Methicillin-Resistant *Stapylococcus aureus* Infections

A 48-year-old man with diabetes and his wife had recurrent methicillin-resistant *Staphylococcus aureus* (MRSA) infections (*30*). Culture of nares samples from the family dog grew mupirocin-resistant MRSA that had a PFGE chromosomal pattern identical to the MRSA isolated from the patient's nares and his wife's wound. The couple reported that the dog routinely slept in their bed and frequently licked their faces. Further recurrence of MRSA infection and nasal colonization in the couple was prevented only after successful eradication of MRSA from the dog's nares.

### Rabies

In many developing countries, being licked by dogs that are rabid or suspected to be rabid is considered to pose a major risk. A survey of rabies exposure among 296 Norwegian missionaries and foreign aid workers traveling abroad showed that of 48 persons for whom postexposure vaccination was recommended, two thirds had only cared for or been licked by the suspected rabid animal (*31*). Rabies remains a problem in Southeast Asia, where many backpackers visit each year. In the early 1990s, foreign travelers (74% of whom were European), who had been in Thailand for an average of 17 days, were surveyed about potential rabies exposure during their visits. Among 1,882 travelers, 1.3% had been bitten and 8.9% had been licked by dogs (*32*). During May–June 2008, another survey of 870 foreign backpackers (median age 25.5 years) in Bangkok, Thailand, found that 3.56% had been licked by a dog (*33*).

### Parasitic Infections

In the United States, the most common parasitic zoonoses associated with dogs are caused by hookworms (*Ancylostoma* spp.) and roundworms (*Toxocara canis*) (*2*). In the Netherlands, prevalent parasitic zoonoses are caused by *Toxocara* spp., *Giardia* spp., *Cryptosporidium* spp., and *Toxoplasma* spp (*1*). Among the ways that toxocariasis can be transmitted to humans, contact with embryonated eggs on a dog's hair coat was recently proposed (*34*). Similarly, a recent study in the Netherlands identified *Toxocara* spp. eggs on the fur of 18 dogs (12.2%) and 2 cats (3.4%) and in the feces of 4 dogs and 1 cat (*1*). That same study found *Giardia* spp. in the feces of 14 dogs and 3 cats and *Cryptosporidium* spp. in feces of 8 dogs and 1 cat (*1*). A case of *Cheyletiella blakei* infection was reported in a 76-year-old woman with pruritic eruption of vesicles and bullous lesions on her trunk and arms (*35*). *Cheyletiella* spp. dermatitis was suspected because of the appearance and distribution of the elementary lesions and because before the eruption, the patient had acquired a cat that sometimes slept in her bed. The diagnosis was confirmed by a veterinary examination and isolation of *C. blakei* from the cat's skin. The patient’s condition resolved after the cat was treated with ivermectin, the household was disinfected with permethrin, and the patient was treated with benzyl benzoate.

## Other Dangers

Another major health hazard can be created by keeping dominant and possessive dogs in a bedroom where young infants are sleeping. An analysis of risk factors associated with nonplay dog bites in Kingston, Jamaica, found that a dog sleeping in a family member’s bedroom was a risk factor for biting (relative risk 2.54, 95% CI 1.4–4.54) (*36*). In a review of fatal dog attacks in the United States during 1989–1994, Sacks et al. (*37*) reported that among 109 dog bite–related deaths, 57% were of children <10 years old and 11 were of a sleeping infant.

## Recommendations

Zoonotic infections acquired by sleeping with a pet are uncommon. However, severe cases of *C. canimorsus* infection or plague in humans have been documented. More zoonotic agents that are transmitted by kissing a pet or being licked by a pet have been identified, especially zoonotic pathogens that are commensal in the oral cavity of carnivores, such as *Pasteurella* spp. and *C. canimorsus.* Because young children are often at higher risk than adults for exposure to zoonotic pathogens, especially when animals are displayed in public settings, the National Association of State Public Health Veterinarians issued specific recommendations (*38*). However, the concerns associated with sharing a bed with pets, being licked by pets, or kissing pets were not addressed in these recommendations. Similarly, although the risk for introduction of zoonotic agents by pets in hospitals or nursing homes has been evaluated (*39*) and recommendations made (*40*), the recommendations do not specifically address the risk for transmission through being licked by, kissing, or even sleeping with a pet.

Our review suggests that persons, especially young children or immunocompromised persons, should be discouraged from sharing their bed with their pets or regularly kissing their pets. Any area licked by a pet, especially for children or immunocompromised persons or an open wound, should be immediately washed with soap and water. Pets should be kept free of ectoparasites (especially fleas), routinely dewormed, and regularly examined by a veterinarian. Preventive measures such as anthelmintic drug intervention for puppies within the first few weeks after birth or, even better, for bitches during the last few weeks of pregnancy, could help prevent most cases of human toxocariasis. Similarly, evaluation of patients with recurrent MRSA colonization or infection or *Pasteurella* spp. infection with no obvious source should prompt queries about any regular contact with pet dogs, particularly in household settings.

## Conclusion

Although uncommon with healthy pets, the risk for transmission of zoonotic agents by close contact between pets and their owners through bed sharing, kissing or licking is real and has even been documented for life-threatening infections such as plague (Table 2). Carriage of ectoparasites or internal parasites is certainly of major concern when it comes to this type of behavior. To reduce such risks, pet owners should seek regular veterinary care for their pets.

**Table 2:** Zoonoses acquired from close contact with pet, 1974–2010*

Zoonosis	Type of pet contact (reference)		
	Sleeping with	Kissing	Being licked by
Plague	Dogs (*7*), Cats (*4*–*6*)	–	–
Chagas disease	Dogs and cats (*8*)	–	–
Cat -scratch disease	Cats, kittens (*10*,*12*); dog (*11*)	–	Kittens (*12*)
Pasteurellosis	Dog (*15*)	Dog (*13*); dogs and cats (*23*,*24*); rabbit (*24*)	Dogs (*16*,*18*,*21*); cats (*14*,*17*,*19*,*20*,*22*); dogs and cats (*14*)
*Capnocytophaga canimorsus* septicemia	Cat (*25*)	–	Dog (*25*–*27*); cat (*25*)
Staphylococcosis	–	–	Dogs (*28*,*29*)
MRSA infection	Dog (*30*)	–	–
Rabies	–	–	Dogs (*31*–*33*)
Toxocariasis	Dogs and cats (*1*)	Dogs and cats (*1*)	Dogs and cats (*1*)
Giardiasis	Dogs and cats (*1*)	Dogs and cats (*1*)	Dogs and cats (*1*)
Cryptosporidiosis	Dogs and cats (*1*)	Dogs and cats (*1*)	Dogs and cats (*1*)
Cheyletiellosis	Dog (*35*)	–	–
Pet bites	Dogs (*36*,*37*)	–	–

*MRSA, methicillin-resistant *Staphylococcus aureus*; –, none reported.

## References

[1] Overgaauw PA, van Zutphen L, Hoek D, Yaya FO, Roelfsema J, Pinelli E, Zoonotic parasites in fecal samples and fur from dogs and cats in the Netherlands. Vet Parasitol. 2009;163:115–22. 10.1016/j.vetpar.2009.03.04419398275

[2] Bingham GM, Budke CM, Slater MR. Knowledge and perceptions of dog-associated zoonoses: Brazos County, Texas, USA. Prev Vet Med. 2010;93:211–21. 10.1016/j.prevetmed.2009.09.01919846225

[3] Westgarth C, Pinchbeck GL, Bradshaw JW, Dawson S, Gaskell RM, Christley RM. Dog–human and dog–dog interactions of 260 dog-owning households in a community in Cheshire. Vet Rec. 2008;162:436–42. 10.1136/vr.162.14.43618390853

[4] von Reyn CF, Weber NS, Tempest B, Barnes AM, Poland JD, Boyce JM, Epidemiologic and clinical features of an outbreak of bubonic plague in New Mexico. J Infect Dis. 1977;136:489–94. 10.1093/infdis/136.4.489908848

[5] Gage KL, Dennis DT, Orloski KA, Ettestad P, Brown TL, Reynolds PJ, Cases of cat-associated human plague in the western US, 1977–1998. Clin Infect Dis. 2000;30:893–900. 10.1086/31380410852811

[6] Lowell JL, Wagner DM, Atshabar B, Antolin MF, Vogler AJ, Keim P, Identifying sources of human exposure to plague. J Clin Microbiol. 2005;43:650–6. 10.1128/JCM.43.2.650-656.200515695659PMC548095

[7] Gould LH, Pape J, Ettestad P, Griffith KS, Mead PS. Dog-associated risk factors for human plague. Zoonoses Public Health. 2008;55:448–54.1848954110.1111/j.1863-2378.2008.01132.x

[8] Gürtler RE, Cécere MC, Rubel DN, Petersen RM, Schweigmann NJ, Lauricella MA, Chagas disease in north-west Argentina: infected dogs as a risk factor for the domestic transmission of *Trypanosoma cruzi.* Trans R Soc Trop Med Hyg. 1991;85:741–5. 10.1016/0035-9203(91)90440-A1801342

[9] Chomel BB, Boulouis HJ, Maruyama S, Breitschwerdt EB. *Bartonella* spp. in pets and effect on human health. Emerg Infect Dis. 2006;12:389–94.1670477410.3201/eid1203.050931PMC3291446

[10] Liao HM, Huang FY, Chi H, Wang NL, Chen BF. Systemic cat scratch disease. J Formos Med Assoc. 2006;105:674–9. 10.1016/S0929-6646(09)60168-616935770

[11] Yamanouchi H, Izumikawa K, Hisamatsu T, Yoshinaga M, Sasaki E, Izumikawa K, A case of *Bartonella henselae* infection from a dog [in Japanese]. Kansenshogaku Zasshi. 2004;78:270–3.1510391010.11150/kansenshogakuzasshi1970.78.270

[12] Zangwill KM, Hamilton DH, Perkins BA, Regnery RL, Plikaytis BD, Hadler JL, Cat scratch disease in Connecticut. Epidemiology, risk factors, and evaluation of a new diagnostic test. N Engl J Med. 1993;329:8–13. 10.1056/NEJM1993070132901028505963

[13] Rhodes M. *Pasteurella multocida* meningitis in a dog lover (or don't kiss pets!). J R Soc Med. 1986;79:747–8.380654810.1177/014107688607901222PMC1290581

[14] Kobayaa H, Souki RR, Trust S, Domachowske JB. *Pasteurella multocida* meningitis in newborns after incidental animal exposure. Pediatr Infect Dis J. 2009;28:928–9. 10.1097/INF.0b013e3181a81f0f19730153

[15] Chikwe J, Bowditch M, Villar RN, Bedford AF. Sleeping with the enemy: *Pasteurella multocida* infection of a hip replacement. J R Soc Med. 2000;93:478–9.1108948410.1177/014107680009300907PMC1298105

[16] Wade T, Booy R, Teare EL, Kroll S. *Pasteurella multocida* meningitis in infancy—(a lick may be as bad as a bite). Eur J Pediatr. 1999;158:875–8. 10.1007/s00431005123210541939

[17] Gullberg RM, Ericson HL, Rearick T, Petrowski S. *Pasteurella multocida* osteomyelitis by a “cat lick.”. Wis Med J. 1997;96:45–6.9197200

[18] Heym B, Jouve F, Lemoal M, Veil-Picard A, Lortat-Jacob A, Nicolas-Chanoine MH. *Pasteurella multocida* infection of a total knee arthroplasty after a “dog lick.”. Knee Surg Sports Traumatol Arthrosc. 2006;14:993–7. 10.1007/s00167-005-0022-516468067

[19] Bryant BJ, Conry-Cantilena C, Ahlgren A, Felice A, Stroncek DF, Gibble J, *Pasteurella multocida* bacteremia in asymptomatic plateletpheresis donors: a tale of two cats. Transfusion. 2007;47:1984–9. 10.1111/j.1537-2995.2007.01421.x17958526

[20] Chun ML, Buekers TE, Sood AK, Sorosky JI. Postoperative wound infection with *Pasteurella multocida* from a pet cat. Am J Obstet Gynecol. 2003;188:1115–6. 10.1067/mob.2003.26612712125

[21] Godey B, Morandi X, Bourdinière J, Heurtin C. Beware of dogs licking ears. Lancet. 1999;354:1267–8. 10.1016/S0140-6736(99)04197-510520644

[22] Nakano H, Sekitani T, Ogata Y, Okazaki H, Tahara T, Hara H. Paranasal sinusitis due to *Pasteurella multocida* [in Japanese]. Nippon Jibiinkoka Gakkai Kaiho. 1993;96:192–6.846388210.3950/jibiinkoka.96.192

[23] Arashima Y, Kumasaka K, Okuyama K, Kawabata M, Tsuchiya T, Kawano K, Clinicobacteriological study of *Pasteurella multocida* as a zoonosis (1). Condition of dog and cat carriers of *Pasteurella*, and the influence for human carrier rate by kiss with the pets [in Japanese]. Kansenshogaku Zasshi. 1992;66:221–4.140208410.11150/kansenshogakuzasshi1970.66.221

[24] Kawashima S, Matsukawa N, Ueki Y, Hattori M, Ojika K. *Pasteurella multocida* meningitis caused by kissing animals: a case report and review of the literature. J Neurol. 2010;257:653–4. 10.1007/s00415-009-5411-019997925

[25] Valtonen M, Lauhio A, Carlson P, Multanen J, Sivonen A, Vaara M, *Capnocytophaga canimorsus* septicemia: fifth report of a cat-associated infection and five other cases. Eur J Clin Microbiol Infect Dis. 1995;14:520–3. 10.1007/BF021134307588825

[26] Dudley MH, Czarnecki LA, Wells MA. Fatal *Capnocytophaga* infection associated with splenectomy. J Forensic Sci. 2006;51:664–6.http://www.ncbi.nlm.nih.gov/sites/entrez?cmd=Retrieve&db=PubMed&list_uids=16696717&dopt=Abstract 10.1111/j.1556-4029.2006.00104.x16696717

[27] Low SC, Greenwood JE. *Capnocytophaga canimorsus*: infection, septicaemia, recovery and reconstruction. J Med Microbiol. 2008;57:901–3. 10.1099/jmm.0.47756-018566152

[28] Kikuchi K, Karasawa T, Piao C, Itoda I, Hidai H, Yamaura H, Molecular confirmation of transmission route of *Staphylococcus intermedius* in mastoid cavity infection from dog saliva. J Infect Chemother. 2004;10:46–8. 10.1007/s10156-003-0281-314991518

[29] Kempker R, Mangalat D, Kongphet-Tran T, Eaton M. Beware of the pet dog: a case of *Staphylococcus intermedius* infection. Am J Med Sci. 2009;338:425–7. 10.1097/MAJ.0b013e3181b0baa919826243

[30] Manian FA. Asymptomatic nasal carriage of mupirocin-resistant, methicillin-resistant *Staphylococcus aureus* (MRSA) in a pet dog associated with MRSA infection in household contacts. Clin Infect Dis. 2003;36:e26–8. 10.1086/34477212522764

[31] Bjorvatn B, Gundersen SG. Rabies exposure among Norwegian missionaries working abroad. Scand J Infect Dis. 1980;12:257–64.700605910.3109/inf.1980.12.issue-4.04

[32] Phanuphak P, Ubolyam S, Sirivichayakul S. Should travelers in rabies endemic areas receive pre-exposure rabies immunization? Ann Med Interne (Paris). 1994;145:409–11.7864502

[33] Piyaphanee W, Shantavasinkul P, Phumratanaprapin W, Udomchaisakul P, Wichianprasat P, Benjavongkulchai M, Rabies exposure risk among foreign backpackers in Southeast Asia. Am J Trop Med Hyg. 2010;82:1168–71. 10.4269/ajtmh.2010.09-069920519619PMC2877430

[34] Lee AC, Schantz PM, Kazacos KR, Montgomery SP, Bowman DD. Epidemiologic and zoonotic aspects of ascarid infections in dogs and cats. Trends Parasitol. 2010;26:155–61. 10.1016/j.pt.2010.01.00220172762

[35] Tsianakas P, Polack B, Pinquier L, Levy Klotz B, Prost-Squarcioni C. *Cheyletiella* dermatitis: an uncommon cause of vesiculobullous eruption [in French]. Ann Dermatol Venereol. 2000;127:826–9.11060386

[36] Messam LL, Kass PH, Chomel BB, Hart LA. The human–canine environment: a risk factor for non-play bites? Vet J. 2008;177:205–15. 10.1016/j.tvjl.2007.08.02017937998

[37] Sacks JJ, Lockwood R, Hornreich J, Sattin RW. Fatal dog attacks, 1989–1994. Pediatrics. 1996;97:891–5.8657532

[38] National Association of State Public Health Veterinarians; Centers for Disease Control and Prevention. Council of State and Territorial Epidemiologists; American Veterinary Medical Association. Compendium of measures to prevent disease associated with animals in public settings, 2009: National Association of State Public Health Veterinarians, Inc. (NASPHV). MMWR Recomm Rep. 2009;58(RR-5):1–21.19407740

[39] Lefebvre SL, Waltner-Toews D, Peregrine AS, Reid-Smith R, Hodge L, Arroyo LG, Prevalence of zoonotic agents in dogs visiting hospitalized people in Ontario: implications for infection control. J Hosp Infect. 2006;62:458–66. 10.1016/j.jhin.2005.09.02516466831

[40] Brodie SJ, Biley FC, Shewring M. An exploration of the potential risks associated with using pet therapy in healthcare settings. J Clin Nurs. 2002;11:444–56. 10.1046/j.1365-2702.2002.00628.x12100640

# Review article: Zoonoses in the Bedroom

**DOI:** 10.3201/eid1702101070

**Authors:** Bruno B. Chomel, Ben Sun

**Affiliations:** Author affiliations: University of California, Davis, California, USA (B.B. Chomel);; California Department of Public Health, Sacramento, California, USA (B. Sun)

## Earning CME Credit

To obtain credit, you should first read the journal article. After reading the article, you should be able to answer the following, related, multiple-choice questions. To complete the questions and earn continuing medical education (CME) credit, please go to **www.medscapecme.com/journal/eid**. Credit cannot be obtained for tests completed on paper, although you may use the worksheet below to keep a record of your answers. You must be a registered user on Medscape.com. If you are not registered on Medscape.com, please click on the New Users: Free Registration link on the left hand side of the website to register. Only one answer is correct for each question. Once you successfully answer all post-test questions you will be able to view and/or print your certificate. For questions regarding the content of this activity, contact the accredited provider, CME@medscape.net. For technical assistance, contact CME@webmd.net. American Medical Association’s Physician’s Recognition Award (AMA PRA) credits are accepted in the US as evidence of participation in CME activities. For further information on this award, please refer to http://www.ama-assn.org/ama/pub/category/2922.html. The AMA has determined that physicians not licensed in the US who participate in this CME activity are eligible for *AMA PRA Category 1 Credits*™. Through agreements that the AMA has made with agencies in some countries, AMA PRA credit is acceptable as evidence of participation in CME activities. If you are not licensed in the US and want to obtain an AMA PRA CME credit, please complete the questions online, print the certificate and present it to your national medical association.

## Article Title: Zoonoses in the Bedroom

**1. Based on the above review by Drs. Chomel and Sun, which of the following statements about transmission of plague by pets living in very close contact to their owners is most likely correct?**A. Transmission of bubonic plague by pet cats has not been reportedB. Transmission of bubonic plague by pet dogs has not been reportedC. Pets infested with infected fleas may transmit plague to their ownersD. Plague cannot be transmitted from infected pets to their owners without biting**2. Your patient is a 6-month-old boy admitted with irritability and fever. Lumbar puncture reveals cerebrospinal fluid profile consistent with meningitis. Upon questioning, the mother admits that while the infant was in a bassinet, she ran to answer the phone, and when she returned to her son, her pet dog was licking his nose and mouth. Based on the above review, which of the following statements is most likely correct?**A. *Pasteurella multocida* has not been linked to meningitisB. Being licked by pets is a common source of human infection by *P. multocida*C. Adults in the household may safely kiss the dog's face without risk of contracting *Pasteurella *spp.D. Sharing the bed with pets has not been reported to cause *Pasteurella *spp. infections**3. Based on the above review, which of the following statements about parasitic zoonoses associated with pets is most likely correct?**A. In the United States, the most common parasitic zoonoses associated with dogs are due to tapewormsB. Transmission of toxocariasis to humans may occur from contact with embryonated eggs on a dog's hair coatC. Aging dogs are most likely to be infected with *Toxocara* spp.D. No measures are currently available to prevent human toxocariasis

## Activity Evaluation

**Table:** 

1. The activity supported the learning objectives.				
Strongly Disagree				Strongly Agree
1	2	3	4	5
2. The material was organized clearly for learning to occur.				
Strongly Disagree				Strongly Agree
1	2	3	4	5
3. The content learned from this activity will impact my practice.				
Strongly Disagree				Strongly Agree
1	2	3	4	5
4. The activity was presented objectively and free of commercial bias.				
Strongly Disagree				Strongly Agree
1	2	3	4	5
